# Astrocytic ATP13A2 modulates neuroinflammatory responses via mitochondrial fission in Parkinson’s disease

**DOI:** 10.3389/fcell.2026.1959910

**Published:** 2026-10-14

**Authors:** Chen Qiao, Huajun Mo, Xiangqi Hu, Tao Dang, Fenglan Xu, Dan Yang, Jiang Ni, Linxiao Wang

**Affiliations:** 1 Department of Pharmacy, Affiliated Hospital of Jiangsu University, Jiangsu University, Zhenjiang, Jiangsu, China; 2 College of Pharmacy, Jiangsu University, Zhenjiang, Jiangsu, China; 3 Department of Pharmacy, Affiliated Hospital of Jiangnan University, Wuxi, Jiangsu, China; 4 The Second People’s Hospital of Changzhou, The Third Affiliated Hospital of Nanjing Medical University, Changzhou, Jiangsu, China

**Keywords:** astrocyte, ATP13A2, mitochondrial fission, neuroinflammation, Parkinson’s disease

## Abstract

**Background:**

Parkinson’s disease (PD) is a global health problem that urgently requires deeper mechanistic insights. Mutations in the ATPase cation transporting 13A2 (*ATP13A2*) gene are linked to autosomal recessive PD, yet the pathological roles of this gene remain incompletely defined. Previous studies on ATP13A2 have focused mainly on its lysosomal functions in neurons, whereas its role in astrocytes has been largely overlooked. Here, we investigated how ATP13A2 affects astrocytic mitochondrial function and contributes to PD pathology.

**Results:**

Using PD mice and *Atp13a2* knockout mice, we found that loss of *Atp13a2* disrupts mitochondrial morphology and dynamics in the substantia nigra, with astrocytes showing both excessive fission and activation. We further found that *Atp13a2* deficiency caused excessive mitochondrial fission, elevated reactive oxygen species (ROS) levels, and promoted extracellular double-stranded DNA release. ELISA measurements revealed significantly increased secretion of IL-1β, IL-6, and TNF-α, indicating increased release of pro-inflammatory cytokines. Importantly, both pharmacological inhibition of fission via Mdivi-1 and *Atp13a2* overexpression restored mitochondrial dynamics homeostasis, suppressed oxidative stress, and reduced cytokine secretion. Collectively, these findings demonstrate that ATP13A2 preserves the mitochondrial fission-fusion balance in astrocytes; its loss breaks this balance, thereby triggering neuroinflammation.

**Conclusion:**

By uncovering previously unrecognized roles of ATP13A2 in regulating mitochondrial fission and subsequent neuroinflammation, this study provides new insights into PD pathogenesis and identifies potential molecular targets for therapy. These findings open fresh avenues for developing more effective strategies to modify PD progression.

## 1 Introduction

Parkinson’s disease (PD), the second most prevalent neurodegenerative disorder worldwide, has seen a near-doubling in prevalence, disability, and mortality over the past 2 decades, with projections indicating further escalation as populations age, posing a growing challenge to global health systems and human quality of life (Outeiro, 2025; Singleton et al., 2025). The neuropathological hallmarks of PD include the progressive degeneration of dopaminergic (DA) neurons in the substantia nigra pars compacta (SNc) and the accumulation of Lewy bodies, composed of misfolded α-synuclein (α-syn) aggregates (Zhou et al., 2024; Blauwendraat et al., 2025). Despite advances in pharmacotherapy, current treatments primarily offer symptomatic relief, failing to halt or reverse the neurodegeneration (Outeiro, 2025).

Mutations in *ATP13A2*, a gene linked to the rare autosomal recessive Kufor-Rakeb syndrome (Ramirez et al., 2006; Vrijsen et al., 2020), have been shown to disrupt lysosomal integrity and polyamine homeostasis. This, in turn, leads to toxic lysosomal accumulation, rupture, and downstream cellular damage (Vrijsen et al., 2020; Li et al., 2024). Beyond lysosomal failure, loss of *Atp13a2* impairs intracellular metal ion homeostasis, triggering mitochondrial stress and compromising autophagic clearance of misfolded α-syn (Fujii et al., 2023). These intertwined disruptions contribute to proteotoxicity, organelle damage, and ultimately, the death of DA neurons.

Mitochondria, the principal energy producers in cells (Shen and Dettmer, 2024), rely on tightly regulated fusion and fission dynamics to sustain synaptic function (Liang et al., 2025; Brooks et al., 2026), calcium buffering, and bioenergetic supply (Marzetti et al., 2025; Schon et al., 2025). Mitochondrial dysfunction in PD leads to reduced ATP production and elevated reactive oxygen species (ROS), which drive oxidative stress, mitochondrial fragmentation, and neuronal apoptosis (Ludtmann et al., 2018; Lurette et al., 2023; Zhang et al., 2024). Aggregated α-syn exacerbates this imbalance by inhibiting fusion and promoting fission (Kamp et al., 2010; Nakamura et al., 2011; Krzystek et al., 2021; Brown et al., 2025). These phenomena have been widely documented in PD patients and α-synucleinopathy models (Gao et al., 2022; Thorne and Tumbarello, 2022; Bhatt et al., 2024). DA neurons of the SNc are particularly vulnerable to such damage because they heavily depend on mitochondrial function for survival (Ludtmann et al., 2018; Bhatt et al., 2024). However, most research has focused on neuronal-intrinsic deficits, largely overlooking the role of astrocytes and their mitochondria. Accumulating evidence indicates that astrocytes actively participate in disease progression, yet how impaired mitochondrial dynamics contribute to pathology within these glial pathways remains an open question.

Astrocytes, the most abundant glial cells in the central nervous system (CNS), are emerging as active contributors to PD pathology (Gradisnik and Velnar, 2023; Favetta and Bubacco, 2025). It has been demonstrated that astrocytes can dynamically upregulate mitochondrial biogenesis during periods of high metabolic demand (Cai et al., 2024; Maiese, 2025; Zufferey et al., 2025), such as proliferation and differentiation. Impaired mitochondrial dynamics and elevated ROS in astrocytes drive their transformation into a neurotoxic reactive state (Bernal and Peterson, 2011; Gorina et al., 2022), as evidenced by reduced phagocytosis, suppressed synaptogenesis, and pro-inflammatory cytokine secretion (Ni et al., 2022). This process has been implicated in the development of neurodegenerative diseases including PD (Mulica et al., 2021; Nguyen et al., 2021; Solano Fonseca et al., 2021).

While over a decade of research has linked loss-of-function of *Atp13a2* to mitochondrial impairment, direct mechanistic evidence demonstrating its role in governing mitochondrial dynamics within astrocytes remains entirely absent. Building on our previous finding that ATP13A2 is highly expressed in astrocytes and modulates neuroinflammation in PD (Qiao et al., 2016), we propose a novel triple synergistic pathogenic model to explain ATP13A2-related PD progression. *Atp13a2* deficiency disrupts lysosomal-mitochondrial homeostasis, precipitating astrocytic mitochondrial dysfunction that exacerbates neuroinflammatory and oxidative stress cascades. In response, activated glial cells generate a neurotoxic microenvironment, creating a self-reinforcing loop that synergistically accelerates DA neuron loss. To validate this hypothesis, we systematically investigated how ATP13A2 balances mitochondrial dynamics, with a particular focus on astrocytes during PD progression. Elucidating this triad is crucial for developing disease-modifying therapies that extend beyond symptomatic relief.

## 2 Materials and methods

### 2.1 Methods

All experimental procedures employed in this study were fully reviewed and approved by the Animal Ethics Committee of Nanjing Medical University (IACUC No. 2008067). All methods were carried out in accordance with the relevant guidelines and regulations. In addition, the present study has been reported in accordance with the ARRIVE guidelines (https://arriveguidelines.org).

### 2.2 Mice

Male C57BL/6J wild-type (WT) mice (3–4 months old, body weight 23–27 g) were used in this study. For this study, all live animal procedures were performed at the animal facility of Nanjing Medical University under the approved protocol (IACUC No. 2008067). Animals were housed under controlled environmental conditions (22 °C ± 2 °C, 50%–60% relative humidity, 12 h light/dark cycle) with *ad libitum* access to food and water. Mice were moved to the laboratory for acclimatization 3 days before the start of the experiment. Because the present study was not designed to address sex-specific mechanisms, female mice were not included. To avoid the interference of biological sex, such as estrogen or estrogen receptors, in the development and phenotypic expression of PD (Antzoulatos et al., 2010; Bourque et al., 2011; Alam et al., 2016), only male mice were used for *in vivo* experiments.

### 2.3 Animal model preparation

#### 2.3.1 Acute MPTP model

Mice were weighed and injected intraperitoneally with 1-methyl-4-phenyl-1,2,3,6 -tetrahydropyridine (MPTP, Sigma, Cat#M0896, 30 mg/kg) at 2-h intervals over four consecutive doses. Following the final administration, animals were euthanized 7 days later for tissue sampling. Control mice received equivalent volumes of sterile saline under identical conditions.

#### 2.3.2 Behavioral analysis

Behavioral testing experiments were performed 24 h after the final drug administration. The open field test was used to evaluate the autonomous behavioral ability of mice. After the mice were acclimatized to the environment in the autonomous activity box for 30 min, the mice were placed in the middle of the open field. The video recording system was turned on to record the trajectory of the mice for 5 min, and the crawling speed was calculated using the open field software. The rotarod test was used to evaluate the motor coordination of mice. Mice were first trained on the rotarod at a speed of 10 rpm for 5 min per day over 2 days. On the test day, the rod accelerated gradually from 2 to 20 rpm over a 5-min period. The latency to fall was recorded for each trial, and the mean of three trials was used for analysis.

The pole test was used to evaluate the motor coordination ability of mice. The experiment was conducted using a wooden pole (1 cm diameter, 50 cm height) with a rough surface, topped by a wooden sphere (1.2 cm diameter, rough surface). The mice were trained once daily for 1 min during the first 2 days. On the test day, each mouse was placed head-upward on top of the sphere. The time to orient head-downward (T-Turn) and the total time to descend to the base of the pole (T-TLA) were recorded. Each mouse underwent three trials, and the shortest times for the two measures were used for analysis.

#### 2.3.3 High-performance liquid chromatography (HPLC) analysis of monoamine concentration

The levels of monoamines and their metabolites were determined by HPLC in conjunction with electrochemical detection (HPLC-ECD). The chromatographic system (Waters) consisted of a 1525 pump, an automatic injector 717 plus, and a Beckman C18 reversed-phase column (ODS-5μm, 4.6 mm, 250 mm). The mobile phase was composed of 75 mM Na_2_HPO_4_·12H_2_O buffer (pH 3.9) containing 0.1 mM EDTA, 2.0 mM octylsodium sulfate, and 10% (vol/vol) methanol, 50 mM citric acid, and 2 mM NaCl, with the final pH adjusted to 4.6. The mobile phase was delivered at a flow rate of 1.0 mL/min. Detection was performed using an amperometric detector (Waters 2465). The applied potential of the glassy carbon electrode was set at +1,000 mV relative to the Ag/AgCl electrode. Quantification was carried out using an external calibration curve, and the final amount in the tissue sample was expressed as mg/g wet tissue for amino acids and ng/g wet tissue for monoamines, respectively.

### 2.4 Reactive oxygen species (ROS) detection

To assess total and mitochondrial ROS production, sections/cells were treated with 10 μM 2′,7′-Dichlorodihydrofluorescein (DCFH-DA, Beyotime, Cat# S0033S) and 5 μM MitoSOX (Thermo Fisher Scientific, Cat# M36008) according to the manufacturer’s instructions.

MitoSOX Red was prepared as a 5 mM stock solution in anhydrous DMSO. Sections were then exposed to 5 µM MitoSOX for 15 min at 37 °C in the dark. Following three washes, fluorescence was visualized by confocal microscopy. Fluorescence intensity was analyzed using ImageJ software from at least three independent experiments, with random fields captured.

For DCFH-DA detection, the probe was diluted 1:1,000 (v/v) in serum-free culture medium. After 48 h of drug treatment or transfection, cells were trypsinized, counted, and pelleted (200,000 cells; 1,000g, 5 min). The pellet was resuspended in 200 μL of diluted DCFH-DA working solution, and incubated at 37 °C for 20 min. Cells were then centrifuged again (1,000 g, 5 min), washed three times by resuspension in serum-free medium and centrifugation. Finally, cells were resuspended and mitochondrial ROS levels were measured by flow cytometry. DCFH-DA was intravenously administered 2 h prior to euthanasia to detect intracellular ROS levels *in vivo*. The midbrain tissues were rapidly extracted and sectioned (20 μm) on a cryostat under red light (λ > 600 nm) to prevent probe photodegradation. Sections were mounted in ProLong Diamond antifade reagent and imaged via confocal fluorescence microscopy. All procedures were performed in darkness except for imaging.

### 2.5 Immunohistochemistry (IHC)

Mice were anesthetized and transcardially perfused with 0.9% saline followed by 4% paraformaldehyde. Brains were removed and post-fixed in 4% paraformaldehyde at 4 °C for 24 h. Brain tissues were dehydrated in 20% sucrose followed by 30% sucrose (each for 3 days) and embedded with OCT compound. Coronal sections (30 μm thick) were cut using a Leica cryostat. Following three PBS washes, the sections were blocked with 5% normal goat serum in PBS for 20 min at room temperature. Subsequently, the sections were incubated with primary antibody against tyrosine hydroxylase (mouse anti-TH, 1:160, Invitrogen, Cat# MA1-24654) at 4 °C for 24 h. After three PBS washes, the sections were incubated with HRP-conjugated goat anti-mouse IgG at room temperature for 1 h. Staining was developed using a DAB substrate solution. After drying, images were captured for analysis. The number of TH^+^ neurons in the SNc was counted using Stereo Investigator, while for the striatum, due to its large area, ten random fields of view were acquired under a ×20 objective, and the mean optical density or percentage area of TH staining was calculated using ImageJ. To enable comparison across experimental groups, all data were normalized to the *Atp13a2* ^+/+^ saline group (set as 1.0).

### 2.6 Immunofluorescence (IF)

For IF, slices or cells were blocked with blocking solution in a humid chamber for 30 min at room temperature, and subsequently incubated with primary antibodies at 4 °C for 24 h. The following primary antibodies were used: mouse anti-TH antibody (1:160, Invitrogen, Cat# MA1-24654), rabbit anti-ATP13A2 antibody (1:1000, Sigma, Cat# A9607), mouse anti-GFAP antibody (1: 800, Sigma, Cat# MAB360), rabbit anti-Drp1 antibody (1:5000, Proteintech, Cat# 12957-1-AP). After washing, sections or cells were incubated with the corresponding secondary antibody for 30 min at room temperature, mounted with an anti-fade mounting medium containing DAPI, coverslipped, and imaged under a fluorescence microscope.

For quantitative analyses involving the SNc, coronal midbrain sections were matched to corresponding coronal levels in a standard C57BL/6 mouse brain atlas (Paxinos and Franklin, 2001, 4th edition), corresponding to bregma approximately −2.92 to −3.80 mm. The anatomical boundaries of the SNc were defined using adjacent serial sections stained for TH, which delineates dopaminergic neurons in this region. A fixed region of interest (ROI) template generated from these TH-defined boundaries was then applied bilaterally to the corresponding matched sections stained for ATP13A2, GFAP, and DAPI. All ROI placement and subsequent quantification were performed by an investigator blinded to genotype and treatment group. This approach ensures that SNc boundaries were consistently established across animals without relying solely on GFAP or DAPI signals.

### 2.7 Nissl staining

The slices containing the desired brain regions were selected. Sections were stained with 0.1% cresyl violet for 1.5–3 min, and the excess dye was removed by rinsing in distilled water for 30 s. Differentiation was performed in 95% ethanol for 30 s, followed by dehydration through a graded ethanol series, clearing in xylene for 30 min, and coverslipping with neutral mounting medium. Sections were air-dried and subsequently examined and photographed under a light microscope.

### 2.8 Transmission electron microscopy (TEM)

Mice were anesthetized, and brain tissues were fixed by transcardial perfusion with a fixative solution containing 2% paraformaldehyde and 3% glutaraldehyde. Tissue blocks (1 mm^3^) of the midbrain substantia nigra pars compacta (SNc) were rapidly dissected based on a mouse brain atlas and immersed in the same fixative for 2 h at 4 °C. Sequentially, the tissue blocks were dehydrated in graded ethanol solutions (70%, 80%, 95% for 2 min each, and 100% for 4 min), followed by anhydrous acetone for 2 min. After embedding in epoxy resin, the ultrathin sections were cut using an ultramicrotome, stained with lead citrate, and then observed and photographed under a transmission electron microscope. Eight randomly selected fields from distinct regions of each midbrain sample were imaged by transmission electron microscopy under identical magnification and frame size. The images were used for quantitative analysis of mitochondrial morphology, including size, area, and structural damage. All imaging and measurements were independently performed by two blinded investigators.

### 2.9 Western blotting (WB)

Brain tissue or cells were harvested and lysed in ice-cold lysis buffer using a sonicator. The lysates were incubated on ice for 30 min and then centrifuged at 12,000 g for 15 min at 4 °C. The supernatant was collected, and protein concentration was measured by a BCA protein assay kit (Beyotime, Cat# P0398M). Aliquots of the supernatants were mixed with protein sample buffer, heated at 95 °C for 10 min, and stored at −80 °C until use. For SDS-PAGE, equal amounts of protein were loaded and separated by electrophoresis at a constant voltage of 80 V for 30 min followed by 110 V for the resolving gel until protein separation was complete. The separated proteins were transferred onto polyvinylidene difluoride (PVDF, Millipore, Cat# IPVH00010) membranes. The membrane was blocked with 5% bovine serum albumin (BSA, Beyotime, Cat# ST025) for 1 h at room temperature. The membranes were then incubated with primary antibodies at 4 °C for 24 h. The following primary antibodies were used: rabbit anti-Drp1 (1:5000, Proteintech, Cat# 129571-AP), rabbit anti-phospho-Ser616 Drp1 (1:1000, Invitrogen, Cat# PA5-64821), rabbit anti-Fis1 (1:5000, Proteintech, Cat# 10956-1-AP), rabbit anti-ATP13A2 (1:1000, Sigma, Cat# A9607), rabbit anti-Mfn1 (1:5000, Proteintech, Cat# 13798-1-AP), mouse anti-Mfn2 (1:5000, Proteintech, Cat# 12186-1-AP), rabbit anti-OPA1 (1:2000, Proteintech, Cat# 27733-1-AP). After washing, the membranes were incubated with appropriate horseradish peroxidase (HRP)-conjugated secondary antibodies for 1 h at room temperature. The protein bands were visualized using an enhanced chemiluminescence (ECL) substrate in a darkroom.

### 2.10 Primary astrocytes culture

Primary astrocytes were isolated from neonatal mice (postnatal day 1–3) using a modified version of established protocols (Zhu et al., 2020). Astrocyte purity was assessed by GFAP immunofluorescence (Supplementary Figure S2).

### 2.11 C8-D1A astrocyte cell line culture and transfection

C8-D1A astrocytes were cultured in high-glucose Dulbecco’s modified Eagle’s medium (DMEM, Gibco, Cat# C11995500BT) containing 10% fetal bovine serum (FBS, Gibco, Cat# 04-001-1ACS) at 37 °C in a humidified atmosphere containing 5% CO2. Cells were seeded in 6-well plates and transfected 24 h later with lentiviral encoding *Atp13a2* knockdown (LV-*Atp13a2*-siRNA) or overexpression (LV-*Atp13a2*-OE). PD cell models were established by exposing cells to 400 μM 1-methyl-4-phenylpyridinium (MPP^+^) for 48 h (Supplementary Figure S3D). Additionally, cells were treated with 10 μM Mdivi-1, a selective mitochondrial fission inhibitor (Supplementary Figure S3E).

All LV-*Atp13a2*-siRNA constructs were purchased from Shanghai Genechem CO., Ltd. The target sequences (5′–3′) were as follows:

LV-*Atp13a2*-siRNA (997-1): 5′-GCC​GAC​CAC​TAC​TAC​TGG​TAT-3′

LV-*Atp13a2*-siRNA (998-1): 5′-GCC​GAA​ACA​CTC​GTT​ATA​GAA-3′

LV-*Atp13a2*-siRNA (999-1): 5′-GCA​CCG​TCT​ACA​GCA​TCA​TCA-3′

LV-NC siRNA: 5′-TTCTCCGAACGTGTCACGT-3′

All LV-*Atp13a2*-OE constructs were synthesized from OBiO Technology (Shanghai) Corp. For the overexpression construct, the full-length mouse Atp13a2 cDNA (GenBank accession NM_029097.3; transcript variant 1) was cloned into the pcSLenti-EF1-EGFP-F2A-Puro-CMV vector under the control of the CMV promoter. The resulting overexpression plasmid was designated as pcSLenti-EF1-EGFP-F2A-Puro-CMV-*Atp13a2*-WPRE. The negative control vector was LV-NC OE (GL181 pcSLenti-EF1-EGFP-F2A-Puro-CMV-MCS-WPRE).

### 2.12 CCK-8 cell viability assay and lactate dehydrogenase (LDH) assay

C8-D1A astrocytes were seeded in 96-well plates and incubated at 37 °C in 5% CO2 for 24 h. After 48 h of drug treatment, the culture medium was replaced with 100 μL serum-free medium containing 10 μL CCK-8 solution (Beyotime, Cat# S0039) per well for 1 h. The absorbance at 450 nm was measured by a microplate reader to calculate the cell viability.

For Lactate dehydrogenase (LDH) assay, C8-D1A cells seeded in 96-well plates were treated with drugs for 48 h. The supernatant was aspirated, and 150 μL of LDH-releasing reagent (Beyotime, Cat# C0019) was added and incubated for 60 min. The plates were then centrifuged at 400 rpm for 5 min. Subsequently, 120 μL of supernatant from each well was transferred to a new 96-well plate, mixed with 60 μL of LDH assay working solution, and incubated for 30 min at room temperature in the dark. Absorbance at 490 nm was measured by a microplate reader, and LDH release was calculated.

### 2.13 Mitochondrial membrane potential assay

Mitochondrial Membrane Potential (ΔΨm) was evaluated using both JC-1 and TMRM staining. JC-1 (Beyotime, Cat# C2003S) was diluted 1:200 in JC-1 buffer. After 48 h of drug treatment, cells were incubated with the JC-1 working solution for 15 min at 37 °C. Coverslips were mounted onto glass slides with DAPI-containing antifade medium and incubated for 10 min in the dark. Fluorescence images were acquired using a fluorescence microscope (Zeiss LSM880). In polarized mitochondria, JC-1 formed aggregates with red fluorescence; upon depolarization, JC-1 remained as monomers with green fluorescence.

For TMRM staining, cells were incubated with 200 nM TMRM (Thermo Fisher, Cat# I34361) in culture medium for 30 min at 37 °C in the dark. ΔΨm was quantified by measuring the mean fluorescence intensity using ImageJ software, and values were normalized to the control group.

### 2.14 RNA sequencing, differential expression analysis, and gene set enrichment analysis (GSEA)

Total RNA was isolated with TRIzol reagent, and its quantity and purity were assessed by Bioanalyzer 2100 and RNA 6000 Nano LabChip Kit. RNA samples with RIN >7.0 were used for library construction. The mRNA was purified from 5 μg total RNA via two rounds of Dynabeads Oligo (dT) enrichment, and then fragmented with Magnesium RNA Fragmentation Module. First-strand cDNA was synthesized by reverse transcription using SuperScript II Reverse Transcriptase. U-labeled second-stranded DNA was generated by *E. coli* DNA polymerase I, RNase H, and dUTP solution. Sequencing was performed on an Illumina Novaseq 6000 (2 × 150 bp paired-end). Raw reads were aligned, and differential expression analysis was performed using DESeq2 statistical software. Significantly dysregulated genes were defined by an absolute fold change of |log_2_FC| ≥ 1.0 and an adjusted *P*-value (FDR) of <0.05. For the heatmap visualization of the selected DEGs in Figure 1C, raw FPKM values were utilized to preserve the absolute transcript abundance of these genes across individual samples without any further transformation. Gene Set Enrichment Analysis (GSEA, version 4.1.0) was subsequently performed using the Molecular Signatures Database (MSigDB) C5 collection. Enrichment scores and *P*-values were calculated accordingly. All bioinformatics analyses were conducted by LC-Bio Technology Co., Ltd.

**FIGURE 1:**
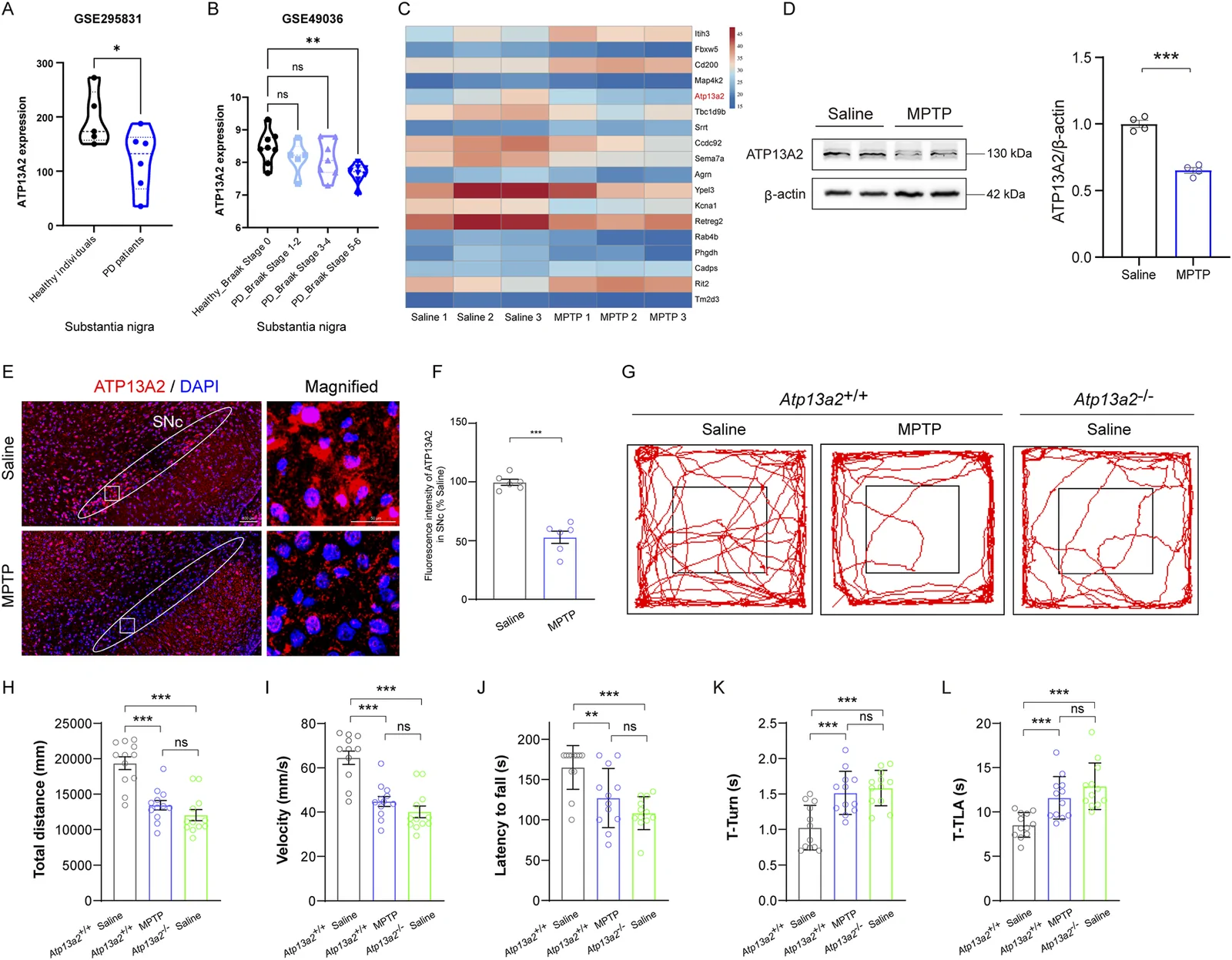
*ATP13A2* dysregulation is associated with PD pathogenesis and drives PD-like motor dysfunction. **(A)** *ATP13A2* mRNA expression levels in the substantia nigra pars compacta (SNc) of PD patients compared with healthy controls from the GEO dataset GSE295831. **(B)** ATP13A2 mRNA expression levels in the SNc across Braak stages (healthy, 1–2, 3–4, and 5–6) based on GSE49036 dataset. **(C)** Transcriptomic analysis of differentially expressed genes (DEGs) between MPTP-treated and saline mice. DEGs were identified by DESeq2 (q < 0.05, fold change >2 or <0.5). The color scale represents raw FPKM (Fragments Per Kilobase of transcript per Million mapped reads) values ranging from 0 to 50, where blue indicates low expression (downregulated genes) and red indicates high expression (upregulated genes) **(D)** Western blot analysis of ATP13A2 protein levels in the midbrain, with β-actin as an internal loading control (n = 4 per group). **(E)** Representative immunofluorescence staining of ATP13A2 (red) in the SNc. Scale bars: 100 μm (left) and 50 μm (right). **(F)** Quantification of ATP13A2 fluorescence intensity (n = 6 per group). **(G)** Representative movement tracesof mice in the open field test (n = 12 per group). **(H)** Total distance traveled in the open field test (n = 12 per group). **(I)** Velocity in the open field test (n = 12 per group). **(J)** Latency to fall in the rotarod test (n = 12 per group). **(K)** Time to turn downward in the pole test (n = 12 per group). **(L)** Time to reach the ground in the pole test (n = 12 per group). For normalization, values were expressed relative to the *Atp13a2* ^+/+^ Saline group mean (set as 100%). Data are presented as mean ± S.E.M. For two-group comparisons **(A,D,F)**, an unpaired two-tailed Student’s t-test was used. For panels with three or more groups **(B,H–L)**, one-way ANOVA followed by Tukey’s *post hoc* test was used. *P < 0.05, **P < 0.01, ***P < 0.001; ns, not significant.

*Additionally, ATP13A2* expression was analyzed in the substantia nigra from PD patients using publicly available datasets. Raw data from GSE295831 and GSE49036 were downloaded from the NCBI GEO database, and human *ATP13A2* expression levels were extracted and analyzed accordingly.

To evaluate the association between *ATP13A2* expression and major neurodegenerative disorders, we performed a transcriptomic correlation analysis using disease-specific gene signatures. Curated gene sets for PD, Alzheimer’s disease (AD), Huntington’s disease (HD), amyotrophic lateral sclerosis (ALS), and prion disease (PrD) were obtained from the MSigDB and the DisGeNET database. For each disease, we calculated the Spearman rank correlation coefficients between *ATP13A2* expression and the mean expression of its signature genes across all samples. In parallel, single-sample gene set enrichment analysis (ssGSEA) scores were derived using the GSVA package in R. Both the correlation coefficients and the ssGSEA scores were normalized to a 0–1 range via min-max normalization to allow direct cross-disease comparison, and were subsequently visualized as a radar chart (Supplementary Figure S1A). Detailed methodology for this analysis is described in the Supplementary Section 1.1.

### 2.15 Quantification of extracellular dsDNA

Double-stranded DNA (dsDNA) in the supernatants was quantified using the Quant-iT PicoGreen dsDNA Assay Kit (Thermo Fisher, Cat# P7589) according to the manufacturer’s instructions. Briefly, DNA standards, positive-control DNA, and samples were incubated with PicoGreen reagent at room temperature in the dark. Fluorescence intensity was measured at an excitation wavelength of 480 nm and an emission wavelength of 520 nm using a fluorescence microplate reader. The dsDNA concentration in each sample was calculated from the standard curve. All procedures were performed under nuclease-free conditions to prevent contamination and DNA degradation. Each sample was assayed in duplicate, and at least three independent experiments were performed. This assay measures total extracellular dsDNA and does not distinguish mitochondrial from nuclear or other origins.

### 2.16 Measurement of inflammatory cytokines by ELISA

Cells were seeded in six-well plates and cultured at 37 °C for 24 h, followed by the indicated treatments for 48 h. Culture supernatants were collected and centrifuged at 1,000 g for 15 min to remove cellular debris. The concentrations of IL-1β, IL-6, and TNF-α were measured using mouse ELISA kits (FCMACS Biotechnology, IL-1β, Cat# FMS-ELM002; IL-6, Cat# FMS-ELM006; TNF-α, Cat# FMS-ELM02B) according to the manufacturer’s instructions. Briefly, standards and samples (100 μL/well) were incubated with 50 μL of biotinylated detection antibody for 2 h at room temperature. After four washes, 100 μL of enzyme conjugate was added and incubated for 30 min. The plates were washed again, followed by incubation with 100 μL of substrate solution for 20 min at room temperature in the dark. The reaction was terminated by adding 100 μL of stop solution, and absorbance was measured at 450 nm using a microplate reader. Cytokine concentrations were calculated from the corresponding standard curves. The IL-1β assay does not specifically distinguish between pro-IL-1β and mature IL-1β.

### 2.17 Statistical analysis

Statistical analyses were performed using GraphPad Prism 9.1. Flow cytometry data were processed with FlowJo software. ImageJ was used for densitometric analysis of protein bands and for relative quantification of IHC and IF images. Data are expressed as Mean ± S.E.M from at least three independent experiments. For comparisons between two groups, an unpaired two-tailed Student’s t-test was used. For comparisons across three or more groups with one independent variable, one-way ANOVA followed by Tukey’s *post hoc* test was applied. For experiments involving two independent factors (e.g., genotype and treatment), two-way ANOVA was applied, followed by Tukey’s *post hoc* tests for pairwise comparisons. *P* < 0.05 indicates that the difference was statistically significant.

## 3 Results

### 3.1 Loss of *Atp13a2* induces PD-related locomotor and coordination impairments

To explore the potential contribution of *ATP13A2* to PD pathology, we examined its expression profile in publicly available clinical transcriptomic datasets. A significant reduction in *ATP13A2* levels was observed in the substantia nigra (SN) of PD patients (GSE295831, Figure 1A). This downregulation was further exacerbated in midbrain derived from individuals at Braak stages 5–6, indicating a progressive decline with disease severity (GSE49036, Figure 1B). RNA sequencing (RNA-seq) transcriptomic analysis of midbrain tissues from MPTP-induced PD model mice identified a total of 18 significantly dysregulated genes, encompassing both up- and downregulated genes in MPTP-treated mice compared with saline controls, as visualized in the heatmap (Figure 1C). Notably, among these transcripts, *Atp13a2* mRNA exhibited a pronounced decrease. To validate this finding, Western blot analysis confirmed a 40.2% reduction in ATP13A2 protein levels in the midbrain of MPTP-induced mice (Figure 1D; values normalized to the Saline group mean, set as 100%), while immunofluorescence (IF) staining revealed a 48.3% decrease in the SNc compared with the saline groups (Figures 1E,F). Reinforcing the disease-specific relevance of these alterations, an integrative transcriptomic analysis encompassing multiple neurodegenerative disorders, conducted via sequencing datasets and presented as radar charts (cross-referenced in Section 2.14), established that *ATP13A2* displays the strongest association with PD among all conditions examined (Supplementary Figure S1A).

Given that motor dysfunction is a primary pathological feature of PD (LeWitt and Chaudhuri, 2020), we assessed voluntary locomotion and motor coordination in *Atp13a2* knockout mice and acute MPTP-treated PD model mice. In the open field test, both experimental groups exhibited a significant attenuation of spontaneous ambulation (Figure 1G); quantitative trajectory analysis confirmed substantial decreases in total distance traveled and mean movement speed compared with wild-type controls (Figures 1H,I). Consistent with these locomotor deficits, the rotarod test revealed a marked shortening of the latency to fall in both the *Atp13a2* knockout group and the MPTP-treated group (Figure 1J), indicating impaired motor coordination and balance. In a parallel assessment, the pole test demonstrated that the time required for head turning and the total descent duration were significantly prolonged in the *Atp13a2* knockout and MPTP-treated groups of mice compared with the wild-type controls (Figures 1K,L). Collectively, these behavioral findings demonstrate that *Atp13a2* knockout induces PD-like motor deficits comparable to those elicited by MPTP administration.

### 3.2 Loss of *Atp13a2* results in midbrain DA neuron loss and striatal dopamine depletion

The primary pathological hallmark of PD is the loss of DA neurons in the midbrain. In this study, we used C57BL/6 J mice to establish the MPTP acute PD model. Nissl staining was performed on midbrain sections to quantify neuronal populations. Consistent with MPTP-induced pathology, *Atp13a2* knockout reduced the number of neurons in the SNc by 41% (Figures 2A,B). Immunohistochemical analysis using tyrosine hydroxylase (TH) staining revealed a 48.9% loss of DA neurons in the SNc (Figures 2C,D). In the striatum, TH immunoreactivity was quantified as fiber/terminal density rather than neuronal number. A 28.8% reduction in striatal TH-positive fiber density was observed in *Atp13a2* knockout mice (Figures 2E,F).

**FIGURE 2:**
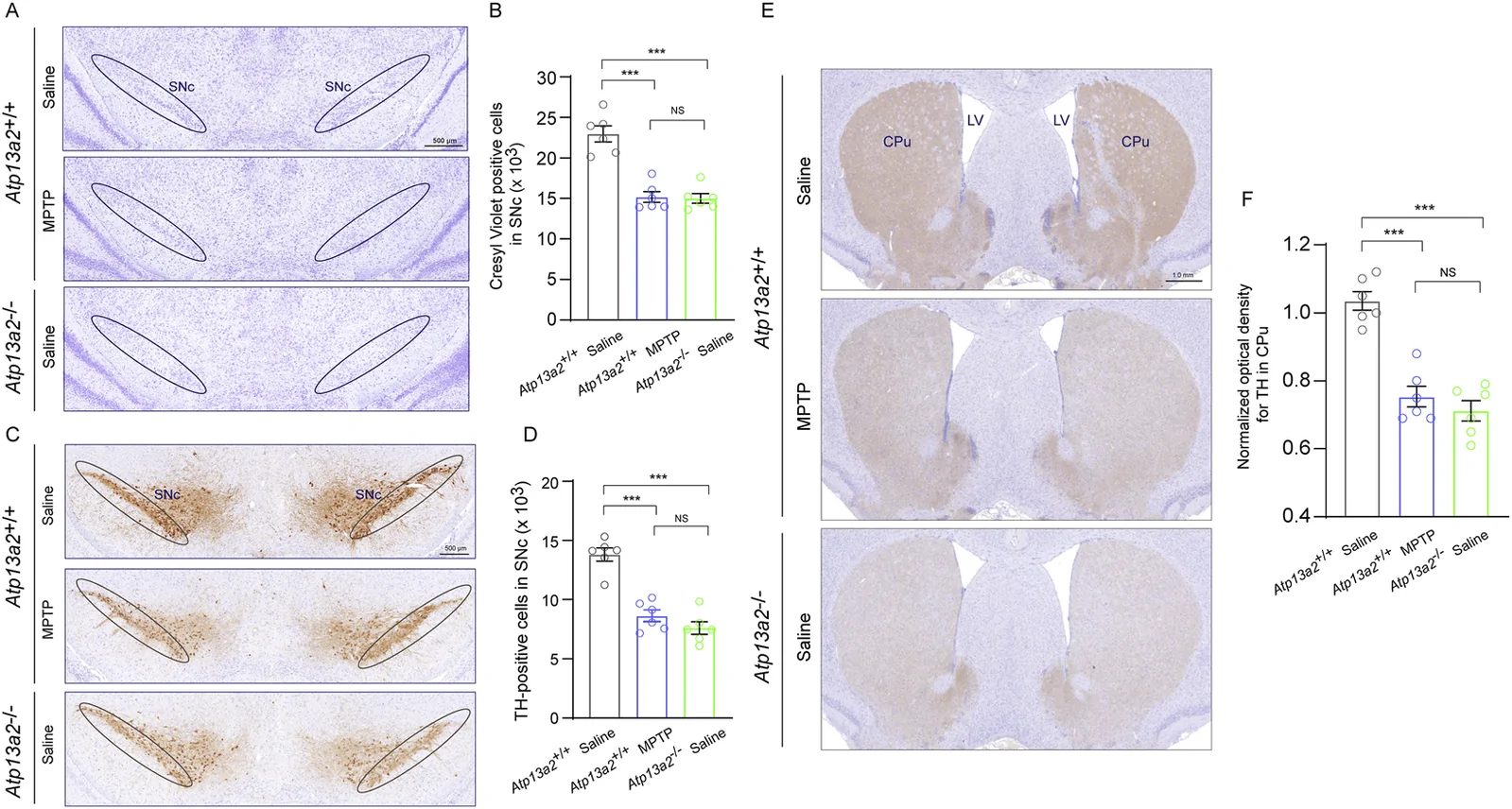
*Atp13a2* deficiency induces loss of midbrain DA neurons in mice. **(A)** Representative Nissl-stained section showing neurons in the SNc of the midbrain. Scale bar: 500 μm. **(B)** Stereological quantification of surviving cells in the SNc (n = 6 per group). **(C)** Immunohistochemical staining of TH-positive neurons in the SNc. Scale bar: 500 μm. **(D)** Stereological counts of surviving TH^+^ neurons in the SNc (n = 6 per group). **(E)** Immunohistochemical staining of TH-positive dopaminergic fibers and terminals in the CPu (striatum). Scale bar: 1 mm. **(F)** Quantification of striatal TH-positive fiber/terminal density (mean optical density) in the CPu, normalized to the *Atp13a2* ^+/+^ Saline group (set to 1.0; n = 6 per group). All percentage changes were calculated relative to the mean of the *Atp13a2* ^+/+^ Saline group, which was set as 100%. Data are presented as mean ± S.E.M., analyzed by one-way ANOVA followed by Tukey’s *post hoc* test. ***P < 0.001; ns, not significant.

To further assess the impact of *Atp13a2* deficiency on the nigrostriatal dopaminergic pathway, striatal levels of dopamine (DA) and its metabolites, 3,4-dihydroxyphenylacetic acid (DOPAC) and homovanillic acid (HVA), were measured by HPLC analysis (Table 1). *Atp13a2* knockout mice exhibited lower levels of DA, DOPAC and HVA in the striatum compared with WT under basal conditions. This finding is largely consistent with the loss of DA neurons in these animals.

**TABLE 1:** Dopamine neurotransmitter levels in the striatum of mice.

Monoamine/metabolite	*Atp13a2* ^+/+^ Saline	*Atp13a2* ^+/+^ MPTP	*Atp13a2* ^-/-^ Saline
**DA**	2.87 ± 0.36	0.45 ± 0.13^*^	1.91 ± 0.30^*^
**DOPAC**	6.51 ± 0.53	1.45 ± 0.25^*^	2.65 ± 0.40^*^
**HVA**	2.04 ± 0.10	0.98 ± 0.20^*^	1.18 ± 0.14^*^

Monoamines and metabolite levels were measured by HPLC with electrochemical detection; DA, dopamine; DOPAC, 3, 4-dihydroxyphenylacetic acid; HVA, homovanillic acid. Data are presented as mean ± S.E.M., from fifteen independent experiments, analyzed by one-way ANOVA followed by Tukey’s *post hoc* test. *P < 0.05 vs the *Atp13a2* ^+/+^ Saline group.

These data indicate that *Atp13a2* knockout leads to the loss of midbrain DA neurons and striatal dopaminergic terminals, contributing to PD progression.

### 3.3 Loss of *Atp13a2* triggers astrocyte activation and aberrant mitochondrial morphology

Based on the transcriptome sequencing results, GSEA analysis revealed significant enrichment of the “Astrocyte development” gene set in MPTP-treated mice, implicating astrocytic developmental pathways in MPTP-induced pathology (Figure 3A). Given the established contribution of reactive astrocytes to neuronal degeneration (Kim and Chung, 2023), we next examined whether *Atp13a2* deficiency affects astrocyte activation in the midbrain. Immunofluorescence staining demonstrated a marked increase in GFAP-positive astrocytes in the SNc of both *Atp13a2* knockout mice and MPTP-treated mice, compared with the *Atp13a2* ^+/+^ Saline controls (Figures 3B,C). Concurrently, Gene Ontology (GO) analysis indicated a potential regulatory role for *Atp13a2* in mitochondrial processes (Figure 3D). To bridge these findings with mitochondrial regulation, double immunofluorescence labeling corroborated that ATP13A2 spatially overlaps with the mitochondrial inner membrane marker TIM23 (Supplementary Figure S1C) and with the mitochondrial fission protein Drp1 (Supplementary Figure S1D), supporting its involvement in mitochondrial dynamics.

**FIGURE 3:**
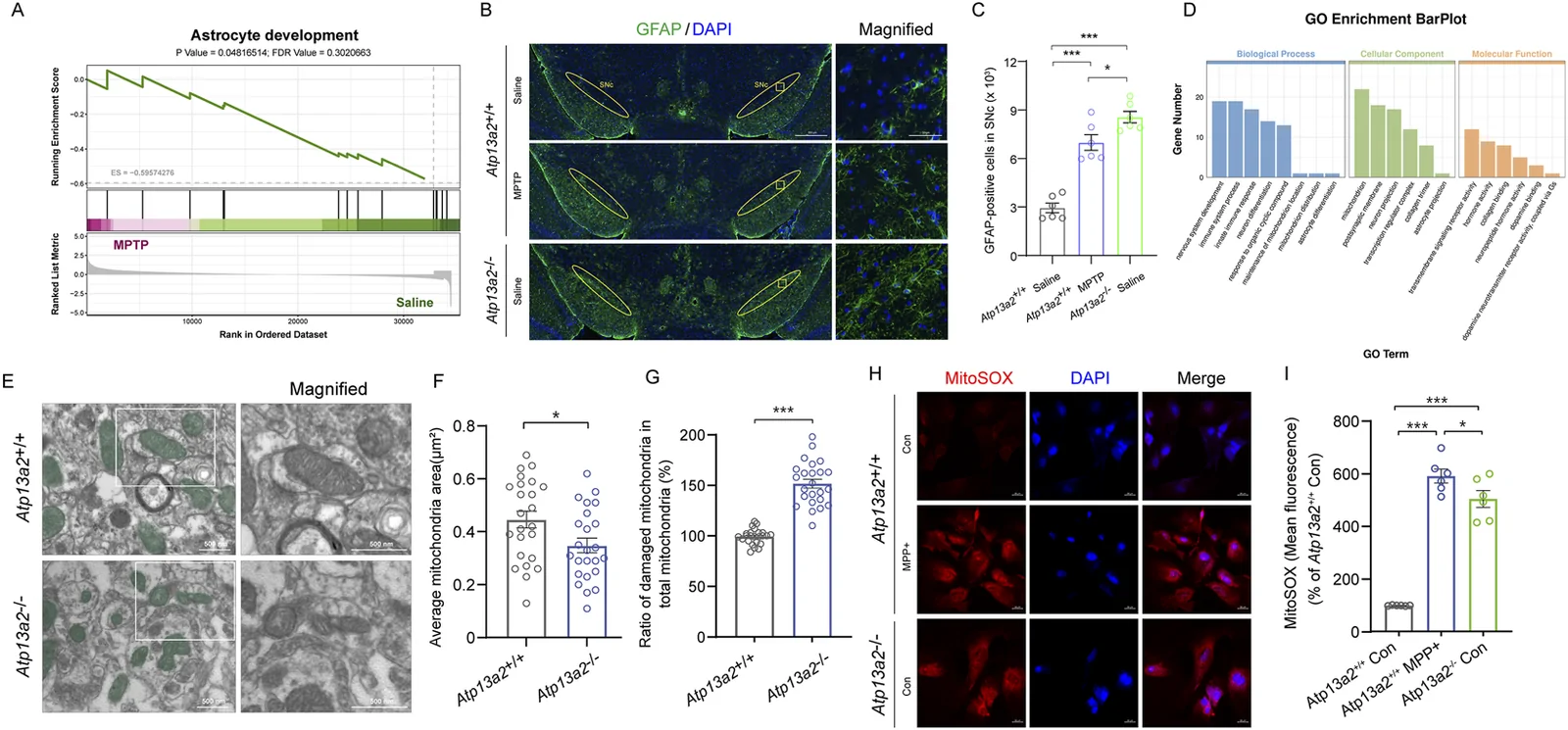
Astrocyte activation and mitochondrial deficits in MPTP and *Atp13a2* deficiency. **(A)** Gene set enrichment analysis (GSEA) identifies “Astrocyte development” as a pathogenic process in MPTP mice (ES = −0.38, *P* = 0.048). **(B)** Representative immunofluorescence staining for GFAP (green) in the midbrain. The yellow outline indicates the SNc region of interest, defined from adjacent TH-stained serial sections and applied bilaterally as a fixed template. Scale bars: 500 μm (left) and 20 μm (right). **(C)** Stereological quantification of GFAP-positive cells (n = 6 per group). **(D)** Gene Ontology (GO) enrichment bar plot revealing the potential regulatory role of *Atp13a2* in mitochondrial processes. **(E)** Representative transmission electron microscopy (TEM) images of midbrain mitochondria from WT and *Atp13a2* knockout mice. Scale bar: 500 nm. **(F)** Quantification of individual mitochondrial area, n = 24 from 3 mice per group. **(G)** Proportion of damaged (swollen or fragmented) mitochondria relative to total mitochondria, n = 24 mitochondria from 3 mice per group. **(H)** Representative fluorescence images of MitoSOX (red) and Hoechst (blue) in primary astrocytes from *Atp13a2* ^+/+^ and *Atp13a2* ^−/−^ mice. Scale bar: 20 μm. **(I)** Quantification of MitoSOX fluorescence intensity in primary astrocytes (n = 6 per group). Data are presented as mean ± S.E.M. For two-group comparisons (F&G), unpaired two-tailed Student’s t-test was used. For panels with three groups (C&I), one-way ANOVA followed by Tukey’s *post hoc* test was used. *P < 0.05, ***P < 0.001; ns, not significant.

We therefore directly examined mitochondrial morphology in the midbrain by transmission electron microscopy (TEM). Compared with the *Atp13a2* ^+/+^ Saline group, *Atp13a2* knockout mice showed pronounced mitochondrial fragmentation, evidenced by a reduced individual mitochondrial area and a higher proportion of damaged mitochondria characterized by swelling and disrupted cristae (Figures 3E–G). To assess whether this morphological disruption was accompanied by oxidative stress, we performed ROS staining on midbrain sections. Notably, *Atp13a*2 deficiency induced widespread oxidative stress, with a substantial portion appearing to originate from non-neuronal sources (Supplementary Figure S1B), prompting a focused investigation on astrocytes. We subsequently cultured primary mouse astrocytes and measured mitochondrial superoxide levels using MitoSOX Red, a probe with higher mitochondrial ROS specificity (Murphy et al., 2022). As a positive control, MPP^+^ treatment induced a marked increase in MitoSOX fluorescence. In primary astrocytes from *Atp13a2* knockout mice, MitoSOX fluorescence intensity was significantly elevated compared with controls (Figures 3H,I), and the increase was comparable to that observed after MPP^+^ treatment. These results indicate that loss of *Atp13a2* disrupts mitochondrial redox homeostasis in astrocytes, consistent with the aberrant mitochondrial morphology observed *in vivo*.

Collectively, these results reveal a pathological cascade in which *Atp13a2* loss leads to aberrant mitochondrial morphology and oxidative stress, thereby promoting proliferative activation of astrocytes in the midbrain. Given the well-established role of reactive astrocytes in secreting cellular danger factors and inducing neuronal cell death (Qian et al., 2023), this *Atp13a2*-dependent astrocyte activation may represent a critical non-cell-autonomous pathological mechanism underlying PD.

### 3.4 Loss of *Atp13a2* induces aberrant mitochondrial fission in midbrain astrocytes

Mitochondria are maintained in a dynamic equilibrium of continuous fusion and fission, which preserves mitochondrial homeostasis and proper intracellular distribution through the regulation of mitochondrial dynamics (Schon et al., 2025). Building on the TEM evidence of mitochondrial abnormalities in the midbrain, we assessed the expression of key mitochondrial dynamics proteins in midbrain tissue lysates. These include the fission-associated proteins p-Drp1, Drp1, and Fis1, and the fusion-associated proteins OPA1, Mfn1, and Mfn2. Western blot analysis revealed significant upregulation of p-Drp1, Drp1, and Fis1 (Figures 4A–D) and downregulation of OPA1, Mfn1, and Mfn2 (Figures 4E–G) in the midbrain of *Atp13a*2 knockout mice compared to controls. Although both p-Drp1(Ser616) and total Drp1 (t-Drp1) were increased, the p-Drp1/t-Drp1 ratio was not significantly altered, indicating that the increase in p-Drp1 largely paralleled the increase in t-Drp1 abundance rather than reflecting preferential Ser616 hyperphosphorylation. These results indicate a disrupted fusion-fission balance, characterized by enhanced fission and impaired fusion, consistent with the abnormal mitochondrial morphology observed via TEM.

**FIGURE 4:**
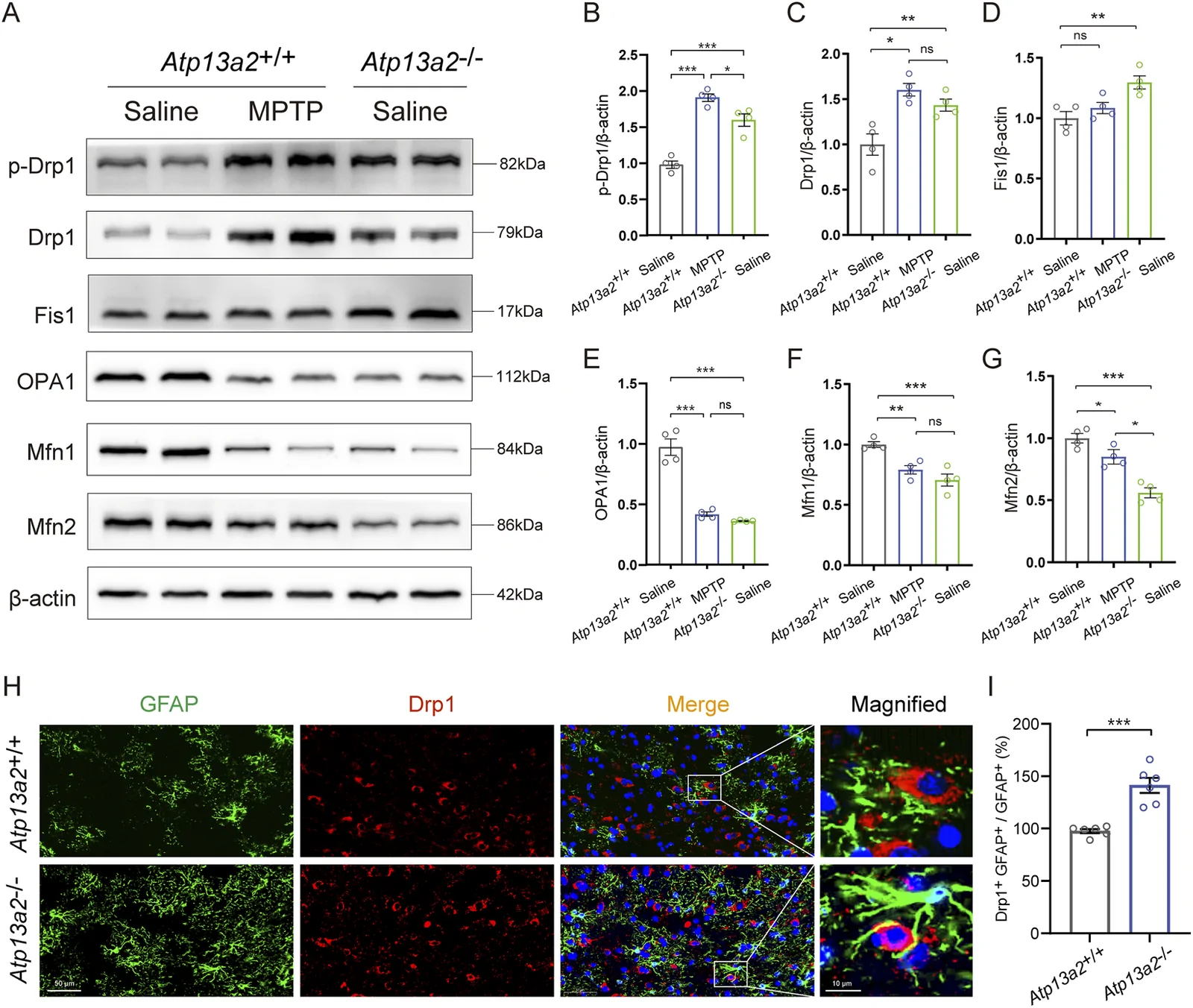
*Atp13a2* deficiency promotes mitochondrial fission and Drp1 accumulation in midbrain astrocytes. **(A)** Western blot analysis of mitochondrial dynamics-related proteins (p-Drp1, Drp1, Fis1, OPA1, Mfn1, Mfn2) in the midbrain (n = 4 per group). **(B)** Quantification of p-Drp1 protein levels. **(C)** Quantification of Drp1 protein levels. **(D)** Quantification of Fis1 protein levels. **(E)** Quantification of OPA1 protein levels. **(F)** Quantification of Mfn1 protein levels. **(G)** Quantification of Mfn2 protein levels. **(H)** Representative immunofluorescence images of the SNc showing Drp1 (red) and GFAP (green) immunoreactivity. Nuclei were counterstained with DAPI (blue). Scale bars: 50 μm (left) and 10 μm (right). **(I)** Percentage of Drp1-positive astrocytes among GFAP-positive cells in the SNc, normalized to the Atp13a2^+/+^ group (set as 100%; n = 6 per group). Data are presented as mean ± S.E.M., analyzed by one-way ANOVA followed by Tukey’s *post hoc* test. For two-group comparisons **(I)**, unpaired two-tailed Student’s t-test was used. *P < 0.05, **P < 0.01, ***P < 0.001; ns, not significant.

Notably, Drp1-associated mitochondrial hyperfission has previously been linked to astrocyte proliferative activation (Joshi et al., 2021). To explore this association between mitochondrial fission and astrocyte activation, we performed double-immunofluorescence labeling of GFAP (an astrocyte marker) and Drp1 in the mouse midbrain. Enhanced GFAP and Drp1 fluorescence was observed in the SNc of *Atp13a*2 knockout mice, with Drp1 upregulation predominantly observed within GFAP-positive astrocytes (Figures 4H,I). These results suggest that *Atp13a2* deficiency is accompanied by enhanced Drp1 accumulation in reactive astrocytes, supporting a potential link between astrocyte activation and aberrant mitochondrial fission.

### 3.5 *Atp13a2* deficiency induces mitochondrial fission and dysfunction in astrocytes

To further verify that *Atp13a2* regulates astrocytic mitochondrial function and contributes to neuroprotection in PD, we performed *in vitro* experiments using the C8-D1A cell line (a widely recognized standard astrocytic model (Zhou et al., 2025)) and an *Atp13a2*-interfering lentiviral system. From three constructed interfering lentiviruses, we selected the sequence with optimal efficacy, LV-*Atp13a2*-siRNA-999, which achieved 50.8% knockdown of ATP13A2 protein in C8-D1A cells (Supplementary Figures S3A–C). This lentivirus was therefore used in all subsequent experiments.

Astrocytes were transduced with LV-*Atp13a2*-siRNA, and Western blot analysis confirmed a significant reduction in ATP13A2 protein expression compared with the LV-negative control (LV-NC) group (Figures 5A,B), thus validating successful gene knockdown. To extend our prior observation of altered mitochondrial dynamics-related proteins in the midbrain of *Atp13a2* knockout mice, we further analyzed these proteins in C8-D1A lysates via Western blot. Consistent with brain tissue findings, *Atp13a2* knockdown astrocytes showed significant upregulation of the fission-associated proteins p-Drp1, Drp1, and Fis1, and downregulation of the fusion protein OPA1 and Mfn2 (Figures 5C–I). Unlike in brain tissue, Mfn1 expression did not differ significantly from controls, a discrepancy potentially attributable to varying degrees of *Atp13a2* knockdown efficiency. As in the midbrain, the p-Drp1/t-Drp1 ratio remained unchanged, indicating that the increase in p-Drp1 reflected the increase in t-Drp1 rather than selective Ser616 hyperphosphorylation.

**FIGURE 5:**
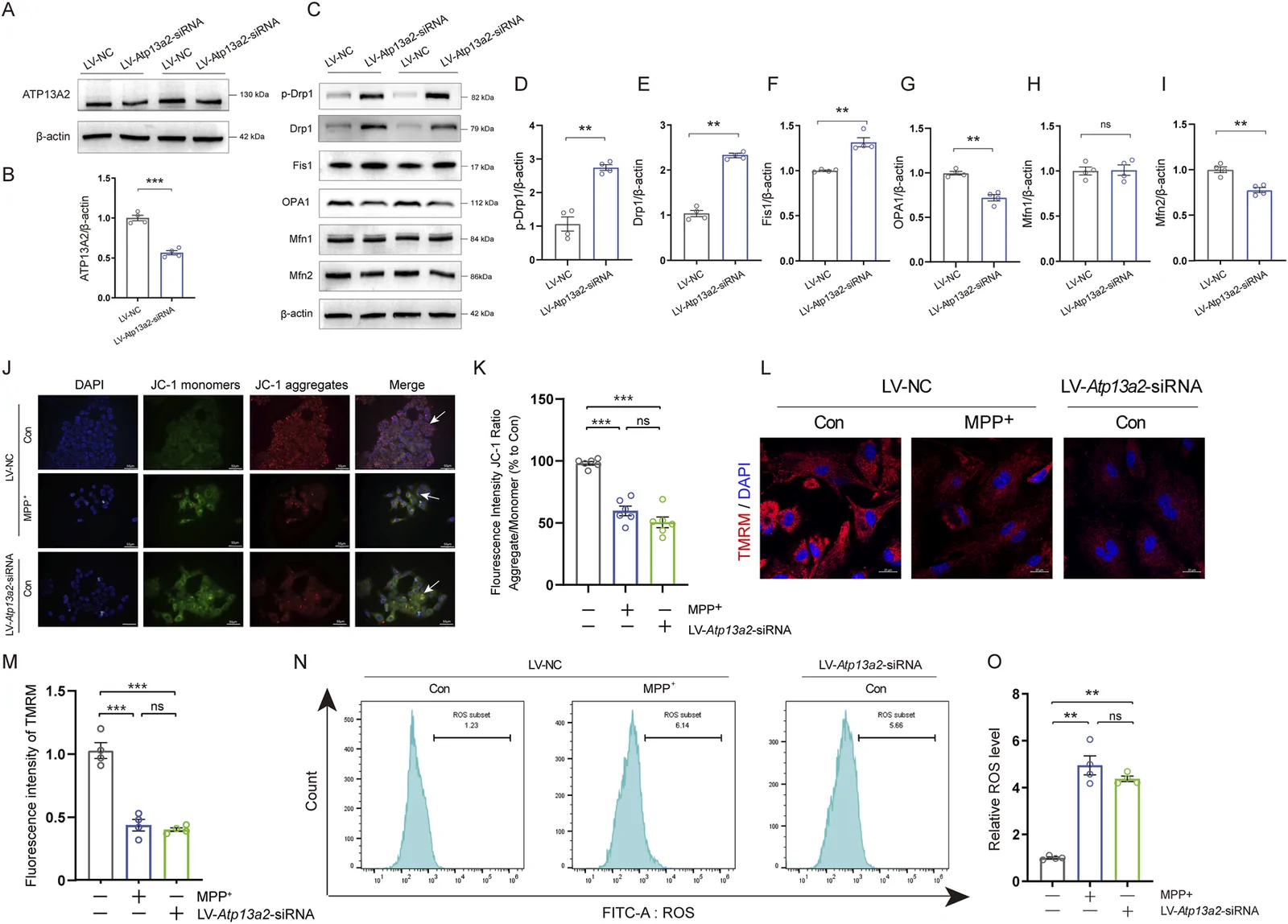
*Atp13a2* deficiency induces mitochondrial dynamics imbalance in C8D1A cells. **(A)** Western blot analysis of ATP13A2 protein levels in C8D1A cells transduced with lentivirus-mediated *Atp13a2* siRNA. **(B)** Quantification of ATP13A2 protein levels. **(C)** Western blot analysis of p-Drp1, Drp1, Fis1, OPA1, Mfn1, and Mfn2 in C8D1A cells (n = 4 independent experiments). **(D)** Quantification of p-Drp1 protein levels. **(E)** Quantification of Drp1 protein levels. **(F)** Quantification of Fis1 protein levels. **(G)** Quantification of OPA1 protein levels. **(H)** Quantification of Mfn1 protein levels. **(I)** Quantification of Mfn2 protein levels. **(J)** Representative fluorescence images of JC-1 staining. Red (J-aggregates) indicates high mitochondrial membrane potential; green (monomers) indicates low membrane potential. Nuclei were counterstained with DAPI (blue). **(K)** Quantification of mitochondrial membrane potential, expressed as the ratio of red-to-green JC-1 fluorescence intensity (n = 6 per group). **(L)** Representative TMRM staining (red) in C8D1A cells. **(M)** Quantification of TMRM fluorescence intensity (n = 4 per group). **(N)** Flow cytometric analysis of ROS levels in C8D1A cells. **(O)** Quantification of ROS levels (n = 4 per group). Data are presented as mean ± S.E.M., analyzed by one-way ANOVA followed by Tukey’s *post hoc* test. For two-group comparisons **(D–I)**, unpaired two-tailed Student’s t-test was used. *P < 0.05, **P < 0.01, ***P < 0.001; ns, not significant.

We next assessed astrocytic mitochondrial function by evaluating mitochondrial membrane potential and ROS production, two cardinal indicators of mitochondrial integrity. Mitochondrial membrane potential (ΔΨm) was first measured by fluorescence microscopy using the JC-1 probe. Under high ΔΨm, JC-1 forms red fluorescent aggregates; under low ΔΨm, it exists as green fluorescent monomers. Both LV-*Atp13a2*-siRNA and MPP^+^ treatment groups exhibited a significant decrease in membrane potential, reflected by reduced red aggregates and increased green fluorescence (Figures 5J,K). Consistent with the JC-1 findings, TMRM staining further confirmed that both MPP^+^ treatment and *Atp13a2* knockdown led to a marked reduction in ΔΨm (Figures 5L,M). As byproducts of mitochondrial energy metabolism (Chen et al., 2026), astrocytic ROS release is known to drive neuroinflammatory responses, while decreased mitochondrial membrane potential correlates with dysfunction. In parallel, intracellular ROS levels were quantified by flow cytometry. Compared with the LV-NC group, both MPP^+^ treatment and *Atp13a2* knockdown significantly elevated ROS production in astrocytes (Figures 5N,O).

Collectively, these results indicate that ATP13A2 depletion in astrocytes triggers excessive mitochondrial fission and functional impairment, supporting a model in which reduced ATP13A2 expression compromises astrocytic homeostasis through dysregulated mitochondrial dynamics.

### 3.6 Mdivi-1 alleviates *Atp13a2* deficiency-induced mitochondrial dysfunction in astrocytes

To determine whether the mitochondrial damage caused by *Atp13a2* knockdown is associated with mitochondrial fission, we treated astrocytes with the mitochondrial fission inhibitor Mdivi-1 (Ye et al., 2025). Mdivi-1 (2.5–10 μmol/L) did not affect astrocyte viability (Supplementary Figure S3E) and, as expected, did not restore ATP13A2 expression in LV-*Atp13a2*-siRNA-transduced cells (Figures 6A,B), consistent with its action downstream of *Atp13a2* loss. We then detected the expression of mitochondrial dynamics-related proteins via Western blot. In *Atp13a2* knockdown astrocytes, Mdivi-1 treatment normalized the expression of Drp1, Fis1, and Mfn2 to control levels. However, the upregulation of p-Drp1 and the downregulation of OPA1 were only partially reversed and remained significantly different from those in the control group (Figures 6C–J). Notably, Mdivi-1 reduced both p-Drp1(Ser616) and t-Drp1, and the p-Drp1/t-Drp1 ratio remained unchanged. Mfn1 expression did not differ significantly across conditions. A similar pattern was observed in MPP^+^-treated astrocytes, in which Mdivi-1 attenuated the increase in p-Drp1 and t-Drp1 and the decrease in Mfn2 (Supplementary Figure S4), confirming that Drp1-associated fission is a common downstream event in both models.

**FIGURE 6:**
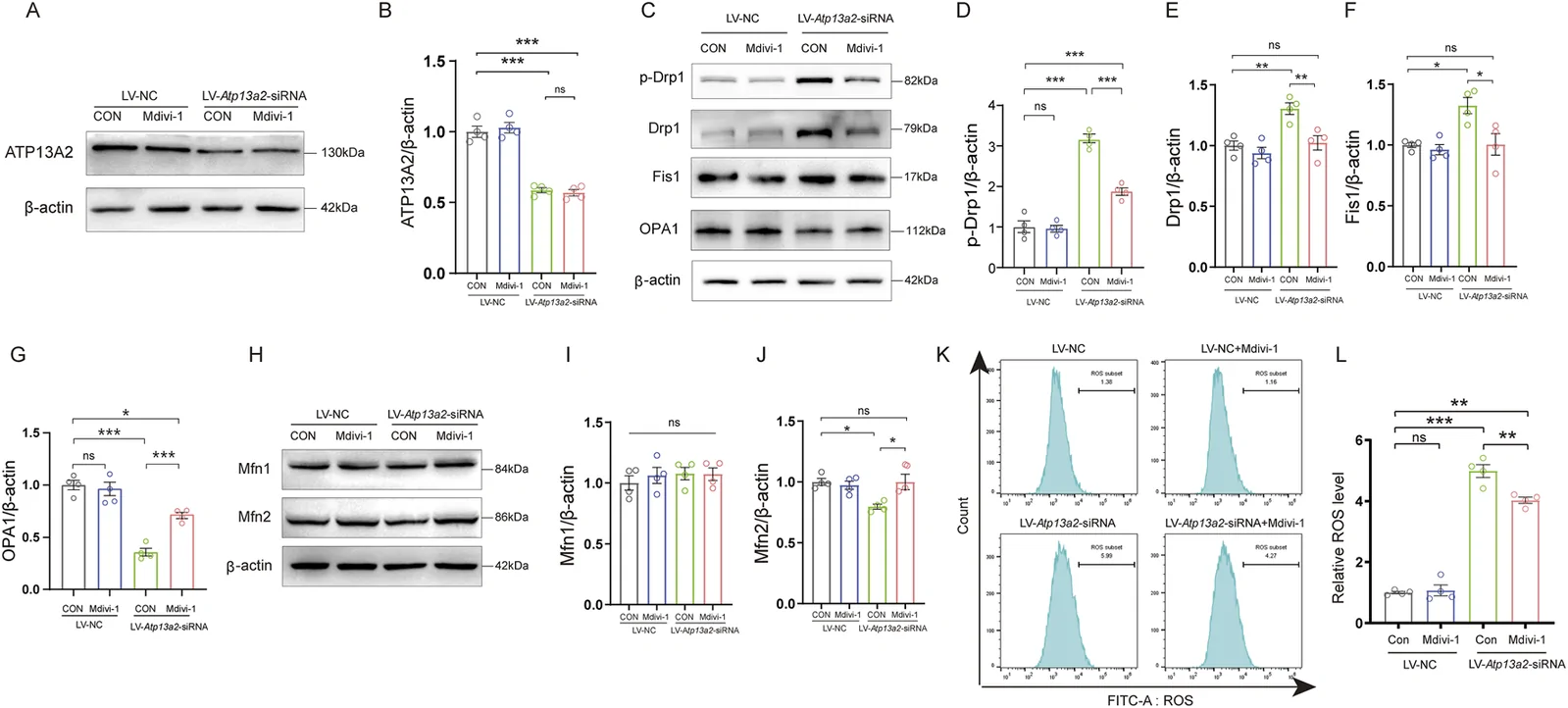
Mdivi-1 ameliorates the mitochondrial dynamics imbalance induced by *Atp13a2* deficiency. **(A)** Western blot analysis of ATP13A2 protein levels (n = 4 per group). **(B)** Quantification of ATP13A2 protein levels. **(C)** Western blot analysis of p-Drp1, Drp1, Fis1, and OPA1 in C8D1A cells (n = 4 per group). **(D)** Quantification of p-Drp1 protein levels. **(E)** Quantification of Drp1 protein levels. **(F)** Quantification of Fis1 protein levels. **(G)** Quantification of OPA1 protein levels. **(H)** Western blot analysis of Mfn1 and Mfn2 protein levels in C8D1A cells (n = 4 per group). **(I)** Quantification of Mfn1 protein levels. **(J)** Quantification of Mfn2 protein levels. **(K)** Flow cytometric analysis of ROS levels in C8D1A cells. **(L)** Quantification of intracellular ROS levels (n = 4 per group). Data are presented as mean ± S.E.M., analyzed by two-way ANOVA followed by Tukey’s *post hoc* test. *P < 0.05, **P < 0.01, ***P < 0.001; ns, not significant.

We next assessed intracellular ROS levels by flow cytometry. Mdivi-1 alone did not alter basal ROS levels, but it significantly reduced the excessive ROS accumulation induced by *Atp13a2* knockdown (Figures 6K,L), although the levels remained above those of control cells. Collectively, these results indicate that pharmacological inhibition of mitochondrial fission can attenuate, but not completely reverse, the mitochondrial dysfunction and oxidative stress caused by *Atp13a2* deficiency.

### 3.7 Restoration of *Atp13a2* expression rescues abnormal mitochondrial fission and attenuates astrocytic inflammation

To further confirm the regulatory role of ATP13A2 in astrocytic mitochondrial dynamics, we restored its expression in *Atp13a2* knockdown astrocytes using a lentiviral overexpression vector (LV-*Atp13a2*-OE). Western blot analysis confirmed that ATP13A2 protein levels were significantly elevated in the LV-*Atp13a2*-siRNA + LV-*Atp13a2*-OE group compared with the LV-*Atp13a2*-siRNA group alone (Figures 7A,B). Notably, genetic restoration of *Atp13a2* significantly reversed the knockdown induced upregulation of p-Drp1(Ser616), Drp1, and Fis1, although these levels remained moderately higher than those in control cells. Conversely, Mfn2 and OPA1 expression was significantly upregulated, whereas Mfn1 levels did not differ from controls (Figures 7C–I). As expected, the p-Drp1/t-Drp1 ratio was not significantly altered under any of these conditions, indicating that ATP13A2 restoration primarily reduced t-Drp1 abundance rather than selectively altering Ser616 phosphorylation.

**FIGURE 7:**
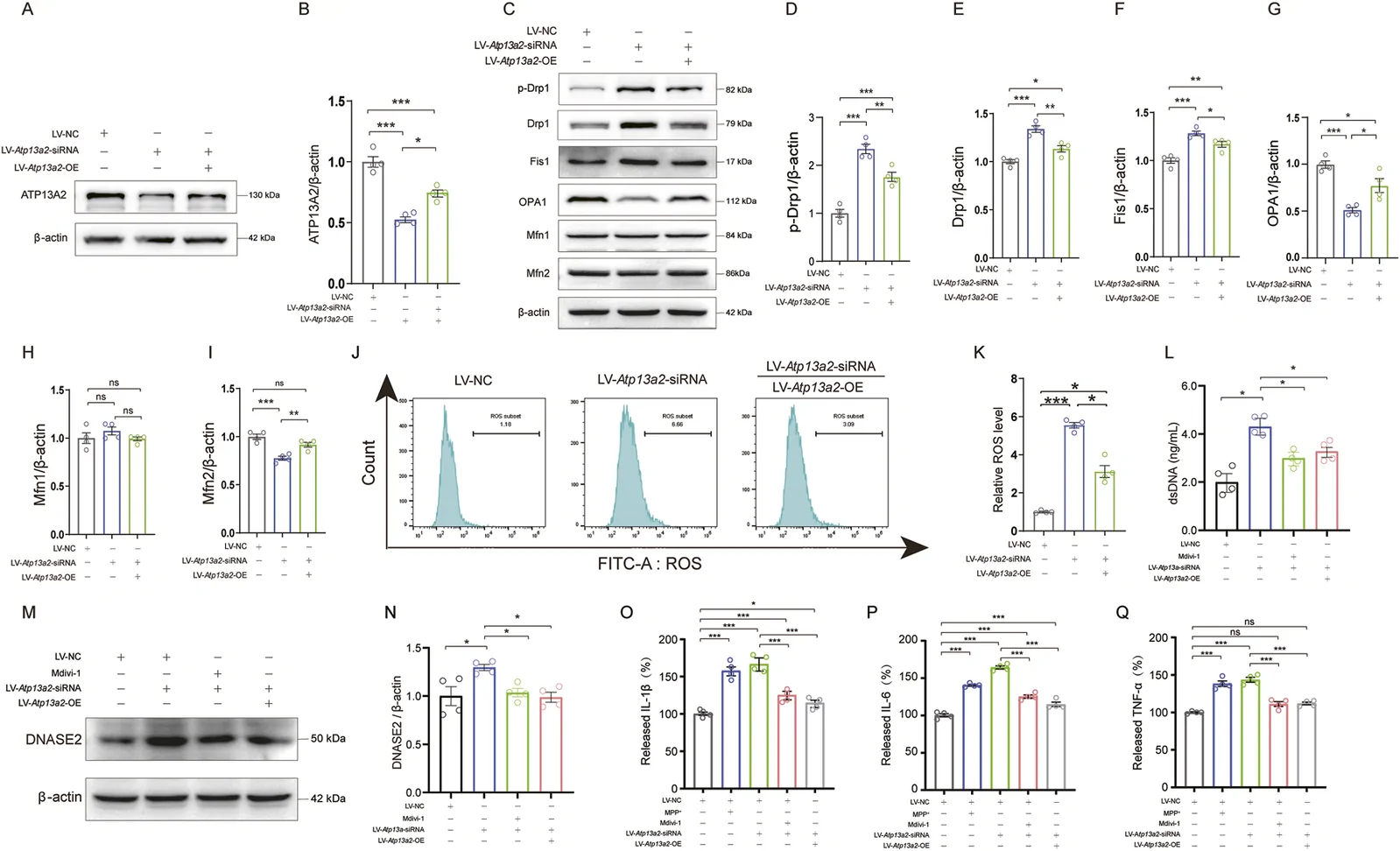
*Atp13a2* overexpression inhibits abnormal mitochondrial fission and reduces inflammatory responses in astrocytes. **(A)** Western blot analysis of ATP13A2 protein levels (n = 4 per group). **(B)** Quantification of ATP13A2 protein levels. **(C)** Western blot analysis of p-Drp1, Drp1, Fis1, OPA1, Mfn1, and Mfn2 in C8D1A cells (n = 4 per group). **(D)** Quantification of p-Drp1 protein levels. **(E)** Quantification of Drp1 protein levels. **(F)** Quantification of Fis1 protein levels. **(G)** Quantification of OPA1 protein levels. **(H)** Quantification of Mfn1 protein levels. **(I)** Quantification of Mfn2 protein levels. **(J)** Flow cytometric analysis of ROS levels in C8D1A cells. **(K)** Quantification of ROS levels (n = 4 per group). **(L)** Quantification of extracellular dsDNA levels in cell culture supernatants. **(M)** Western blot analysis of DNASE2 protein levels (n = 4 per group). **(N)** Quantification of DNASE2 protein levels. **(O)** Quantification of released IL-1β levels in cell culture supernatants. **(P)** Quantification of released IL-6 levels in cell culture supernatants. **(Q)** Quantification of released TNF-α levels in cell culture supernatants. Data are presented as mean ± S.E.M. For panels **A–K**, one-way ANOVA followed by Tukey’s *post hoc* test was used. For panels **L–Q**, two-way ANOVA followed by Tukey’s *post hoc* test was used. *P < 0.05, **P < 0.01, ***P < 0.001; ns, not significant.

We next assessed intracellular ROS by flow cytometry. *Atp13a2* overexpression significantly attenuated the excessive ROS production induced by *Atp13a2* knockdown, with ROS levels in the rescue group (LV-*Atp13a2*-siRNA + LV-*Atp13a2*-OE) being markedly lower than those in the knockdown group (LV-*Atp13a2*-siRNA) (Figures 7J,K). To validate these findings in a pathophysiological context, we also examined the effect of *Atp13a2* overexpression in MPP^+^-treated C8-D1A cells. Overexpression of *Atp13a2* partially restored its expression, which was suppressed by MPP^+^ (from 52% to 74% of control levels; Supplementary Figures S5A,B), and effectively reversed the MPP^+^-induced upregulation of p-Drp1, Drp1, and Fis1, as well as the downregulation of Mfn2 (Supplementary Figures S5A–F). Consistent with these changes, *Atp13a2* overexpression also rescued the MPP^+^-induced loss of mitochondrial membrane potential, as shown by TMRM staining (Supplementary Figures S5G,H). Notably, *Atp13a2* overexpression was more effective than Mdivi-1 in restoring Mfn2 expression, indicating that genetic reconstitution of *Atp13a2* achieves a more comprehensive correction of the fission-fusion imbalance.

Given that excessive mitochondrial fission has been associated with extracellular dsDNA release and downstream inflammatory cascades, we next sought to determine whether Mdivi-1 or *Atp13a2* overexpression could attenuate this process. *Atp13a2* knockdown significantly increased DNASE2 protein levels (Figures 7L,M) and elevated dsDNA release into the supernatant (Figure 7N). Both Mdivi-1 treatment and *Atp13a2* overexpression markedly suppressed the DNASE2 upregulation and extracellular dsDNA release induced by *Atp13a2* deficiency (Figures 7L–N). Furthermore, the excessive release of pro-inflammatory cytokines, including IL-1β, IL-6, and TNF-α, triggered by *Atp13a2* knockdown was similarly reversed by both treatments (Figures 7O–Q).

Collectively, these findings demonstrate that restoration of ATP13A2 expression counteracts Drp1-associated mitochondrial hyperfission, reduces intracellular ROS accumulation, and limits DNA release and inflammatory responses in astrocytes. This reveals a mechanism by which ATP13A2 sustains mitochondrial dynamic homeostasis to delay astrocyte-driven neuroinflammation and confer neuroprotection in PD.

## 4 Discussion

This study identifies a previously unrecognized role for ATP13A2 in regulating mitochondrial dynamics in astrocytes, thereby contributing to PD pathology. ATP13A2 is classically characterized as a transmembrane P5-type ATPase that maintains metal ion homeostasis and lysosomal autophagy (Ramirez et al., 2006; Dang et al., 2022). However, most prior evidence derives from non-neuronal or non-PD models, leaving open the possibility of cell-type- or disease-stage-specific functions. Our findings reveal that loss of *Atp13a2* leads to severe mitochondrial ultrastructural defects in the midbrain. Although TEM cannot assign these abnormalities to a specific cell type, behavioural and histological analyses further showed that *Atp13a2* knockout causes neurodegeneration accompanied by excessive mitochondrial fission in GFAP-positive astrocytes. Pharmacological inhibition of mitochondrial fission using Mdivi-1 (Ye et al., 2025), or overexpression of *Atp13a2*, reversed these effects and preserved mitochondrial dynamic homeostasis, suggesting a functional link between ATP13A2 and astrocytic mitochondrial fission.

Astrocytes are the most abundant glial cells in the brain and support synaptic maintenance and neuronal metabolism (Gradisnik and Velnar, 2023). Growing evidence indicates that astrocytic mitochondrial impairment is an early event in PD (Liu et al., 2018; Kuter et al., 2019). Moreover, the PD-linked kinase PINK1 phosphorylates its substrates predominantly in astrocytes rather than neurons (Barodia et al., 2019). Consistent with the “mitochondria-astrocyte-neuron triad hypothesis” (Walecha and Luthra, 2025), our study provides several lines of evidence for astrocytic involvement: transcriptomic changes indicating altered astrocyte development and mitochondrial function (Figures 3A,D); prominent astrocyte hyperproliferation and activation *in vivo* (Figures 3B,C); non-tyrosine hydroxylase-derived ROS in the midbrain (Supplementary Figure S1B); and direct cell-autonomous mitochondrial ROS elevation in primary *Atp13a2* knockout astrocytes (Figures 3H,I). These findings support the concept that astrocytic mitochondrial dysfunction contributes to PD progression.

In midbrain tissue, ATP13A2 showed spatial overlap with the inner membrane marker TIM23 and with the fission protein Drp1 (Supplementary Figures S1C,D). Because these experiments lacked an astrocyte-specific marker, this observation is interpreted solely as a tissue-level mitochondrial association. This interpretation is consistent with previous reports showing ATP13A2 proximity to TOM20 (den Brave et al., 2021) and its translocation to the outer mitochondrial membrane upon depolarization (Hatori et al., 2022). To determine whether *Atp13a2* loss affects mitochondrial fission specifically in astrocytes, we quantified Drp1 immunofluorescence within GFAP-positive cells and observed a significant increase upon *Atp13a2* loss (Figure 4H), supporting enhanced astrocytic mitochondrial fission. It is important to note that fluorescence colocalization indicates spatial proximity only and does not demonstrate direct physical interaction or regulatory coupling. The precise subcellular site of ATP13A2 action, whether at membrane contact sites, near the outer mitochondrial membrane, or within a dynamic organelle interaction network, requires future high-resolution imaging and ultrastructural studies.

Mitochondrial dysfunction is central to PD pathogenesis (Brooks et al., 2026), yet no effective mitochondria-targeted therapy currently exists (Schon et al., 2025). Here, Mdivi-1, a pharmacological inhibitor of mitochondrial division (Ye et al., 2025), suppressed Drp1 upregulation and attenuated the astrocytic mitochondrial damage caused by *Atp13a2* knockdown, without restoring ATP13A2 expression. Conversely, astrocytic overexpression of *Atp13a2* also exerted its protective effects (Figure 7). This suggests that ATP13A2 may function upstream of mitochondrial fission. Notably, ATP13A2 deficiency increased not only Drp1 but also Fis1, another key regulator of mitochondrial fission, suggesting that the observed mitochondrial fragmentation may involve coordinated alterations in multiple fission-related proteins rather than a single Drp1-dependent pathway. Accordingly, the phenotype is more accurately described as Drp1-associated excessive fission rather than strictly Drp1-mediated. Because multiple mitochondrial dynamics proteins were altered, single-gene knockdown of *Drp1* or *Fis*1 alone may yield incomplete rescue; this rationale guided our use of pharmacological inhibition despite its known Drp1-independent effects of Mdivi-1, including complex I suppression (Bordt et al., 2017; Ruiz et al., 2018; Marx et al., 2024). Therefore, our data support an association between *Atp13a2* deficiency and enhanced Drp1-related fission within a broader disturbance of mitochondrial dynamics. Whether ATP13A2 directly interacts with Drp1, Mfn1, Mfn2, or Fis1 remains unresolved and warrants future investigation.

Our previous work established an anti-neuroinflammatory function for ATP13A2 in astrocytes but focused solely on lysosomal pathways (Qiao et al., 2016). Because lysosome-targeted interventions alone cannot fully reverse ATP13A2 loss-induced damage (van Veen et al., 2020), additional regulatory mechanisms are likely. Although ATP13A2 may indirectly affect mitochondria via lysosomal metal ion modulation (Park and Sue, 2017; Croucher and Fleming, 2023), direct regulation has not been documented. Given the established role of ATP13A2 in autophagic-lysosomal pathways, the mitochondrial phenotypes observed here could be secondary to lysosomal dysfunction. Our data do not exclude this possibility. Thus, an association with mitochondria-lysosome contact sites remains an alternative interpretation, potentially involving a cross-organelle regulatory model in which ATP13A2 facilitates lysosome-mitochondria crosstalk.

Beyond these organelle-level considerations, the inflammatory readouts used in the present study also require cautious interpretation. Although our ELISA data demonstrate increased release of IL-1β, IL-6, and TNF-α from *Atp13a2*-deficient astrocytes, they do not identify the specific inflammatory signaling pathway responsible for this response. The IL-1β assay measures IL-1β in culture supernatants and does not distinguish between pro-IL-1β and mature IL-1β. Similarly, the PicoGreen assay measures total extracellular dsDNA and does not establish its mitochondrial origin. These findings should therefore be interpreted as evidence of increased pro-inflammatory cytokine release and extracellular dsDNA release rather than definitive activation of a particular inflammasome or DNA-sensing pathway. Future studies combining mtDNA-specific qPCR or co-labeling of dsDNA with mitochondrial markers, together with caspase-1/NLRP3 or cGAS-STING readouts (Zhou et al., 2011; Wu et al., 2013), will be required to define the mechanism linking mitochondrial fission and extracellular dsDNA release to cytokine production.

In addition to these assay-level limitations discussed above, the choice of PD model also warrants consideration. The MPTP/MPP^+^ model was selected because of its direct relevance to the mitochondrial focus of this study. MPTP crosses the blood-brain barrier and is converted to MPP^+^, which is preferentially taken up by dopamine transporter-expressing dopaminergic neurons and can also be taken up by astrocytes, and inhibits mitochondrial complex I, leading to increased mitochondrial ROS, impaired bioenergetics, and mitochondrial fragmentation (Duty and Jenner, 2011; Bove and Perier, 2012). These features align with the astrocytic mitochondrial phenotype observed upon *Atp13a2* loss. However, this acute/subacute toxin model does not fully recapitulate the chronic, age-dependent, and α-synuclein-linked pathology of sporadic PD. Validation in complementary models, such as 6-OHDA, rotenone, or α-synuclein-based models, will therefore be important to assess the generalizability of our findings (Duty and Jenner, 2011). Beyond model-specific considerations, because this study employed a global *Atp13a2* knockout model, the *in vivo* findings should be interpreted within the context of systemic *Atp13a2* deficiency rather than as definitive evidence of astrocyte-specific causality. To strengthen the cellular relevance, we used primary mouse astrocytes and astrocyte cell lines, which supported the conclusion that ATP13A2 contributes to the maintenance of astrocytic mitochondrial homeostasis. Nevertheless, future studies using astrocyte-specific conditional knockout models, such as GFAP-Cre or Aldh1L1-Cre strategies, will be necessary to precisely define the causal contribution of astrocytic ATP13A2 to mitochondrial dysfunction and neurodegenerative progression *in vivo*.

In summary, our study uncovers a previously unrecognized mechanism by which ATP13A2 regulates astrocytic mitochondrial fission, preserves mitochondrial dynamics, and modulates neuroinflammation in PD models. These findings position ATP13A2-related pathways as a promising therapeutic target to slow PD progression.

## Data Availability

The datasets presented in this study can be found in online repositories. The NCBI Gene Expression Omnibus (GEO): GSE346387.

## Glossary

PD: Parkinson’s disease
ATP13A2: adenosine triphosphatase cation transporting 13A2
DA: dopamine
SNc: substantia nigra pars
α-syn: alpha-synuclein
ROS: reactive oxygen species
WT: wild-type
MPTP: 1-methyl-4-phenyl-1,2,3,6-tetrahydropyridine
HPLC: high-performance liquid chromatography
DHE: dihydroethidium
PBS: phosphate-buffered Saline
TH: tyrosine hydroxylase
DAPI: 4′,6-diamidino-2-phenylindole
GFAP: glial fibrillary acidic protein
Drp1: dynamin-related protein 1
TEM: transmission electron microscopy
SDS-PAGE: sodium dodecyl sulfate-polyacrylamide gel electrophoresis
PVDF: polyvinylidene fluoride
Fis1: mitochondrial fission protein 1
Mfn: Mitofusin
TOM20: translocase of outer membrane 20
LV-ATP13A2-siRNA: lentiviral-encapsulated ATP13A2 knockdown plasmid
LV-ATP13A2-OE: lentiviral-encapsulated ATP13A2 overexpression plasmid
CCK-8: cell counting kit-8
LDH: Lactate dehydrogenase
DCFH-DA: 2′,7′-dichlorodihydrofluorescein diacetate
JC-1: 5,5′,6,6′-tetrachloro-1,1′,3,3′-tetraethylbenzimidazolylcarbocyanine iodide
GSEA: gene set enrichment analysis
KEGG: kyoto encyclopedia of genes and genomes
ANOVA: Analysis of Variance
HVA: homovanillic acid
CPu: Caudate Putamen
DOPAC: 3,4-dihydroxyphenylacetic acid
LV-NC: LV-Negative control
Mdivi-1: Mitochondrial Division Inhibitor 1
MPP+: 1-Methyl-4-phenylpyridinium
