# Supplementary material for: Astrocytic ATP13A2 modulates neuroinflammatory responses via mitochondrial fission in Parkinson’s disease
Source: Front Cell Dev Biol. 2026 Oct 14;14:1959910. doi: 10.3389/fcell.2026.1959910 (PMC13713819; doi:10.3389/fcell.2026.1959910)
Supplement: Supplementary file 1 [file Supplementaryfile1.docx]

Supplementary Material

# 1 Extended Methodology for Supplementary Figures

## 1.1 Correlation analysis between *ATP13A2* and neurodegenerative disease gene signatures

To evaluate the association between *ATP13A2* expression and major neurodegenerative disorders, we performed correlation analyses using disease‑specific gene signatures. Curated gene sets for Parkinson’s disease (PD), Alzheimer’s disease (AD), Huntington’s disease (HD), amyotrophic lateral sclerosis (ALS), and prion disease (PrD) were obtained from the Molecular Signatures Database (MSigDB; including KEGG and Gene Ontology gene sets) and the DisGeNET database. For each disease, the mean expression of its corresponding signature genes was calculated from the same RNA‑seq dataset. Spearman’s rank correlation coefficients between *ATP13A2* expression and these mean signature values were then computed across all samples. As a complementary approach, single‑sample gene set enrichment analysis (ssGSEA) scores for each disease gene set were derived using the GSVA package in R. Both the correlation coefficients and the ssGSEA enrichment scores were normalized to a 0-1 range via min–max normalization, allowing direct cross‑disease comparison. The normalized values were visualized as a radar chart, with each axis representing one neurodegenerative disease.

# 2 Supplementary Figures

## 2.1 Supplementary Figure 1

**Supplementary Figure 1. ATP13A2 is linked to mitochondria in PD.** (A) Radar chart showing the association of *ATP13A2* with neurodegenerative disorders. (B) Representative immunofluorescence staining for TH (green) and DCFH‑DA fluorescence (red) in the midbrain. Scale bar: 20 μm. Double immunofluorescence staining of mouse midbrain sections showing spatial overlap (yellow) of ATP13A2 (red) with TIM23 (green, C) or Drp1 (orange, D), indicating a mitochondrial association of ATP13A2 at the tissue level. Scale bars: 50 μm (left) and 10 μm (right).

## 2.2 Supplementary Figure 2

**Supplementary Figure 2.** **Purity validation of primary astrocytes.** (A) Representative immunofluorescence images of primary astrocytes stained with GFAP (astrocyte marker, green) and DAPI (nuclei, blue). Scale bar: 100 μm. (B) Percentage of GFAP⁺ cells in primary cultures.

## 2.3 Supplementary Figure 3

**Supplementary Figure 3.** **Optimization of lentivirus-mediated *Atp13a2* knockdown and MPP⁺/Mdivi‑1 treatment conditions in C8‑D1A cells.** (A) C8‑D1A cells were transduced with lentivirus carrying GFP‑tagged interfering RNA at the indicated titers. A titer of 5 × 10⁸ TU/ml achieved approximately 50% knockdown efficiency, as assessed by the relative reduction of the target protein or mRNA. (B) Three independent *Atp13a2*‑targeting siRNA sequences (LV‑*Atp13a2*‑siRNA) were evaluated for their knockdown efficiency. (C) Among the tested sequences, LV‑*Atp13a2*‑siRNA‑999 most effectively downregulated ATP13A2 expression and was selected for subsequent experiments. (D) C8‑D1A cells were treated with the indicated concentrations of MPP⁺ for different durations, and LDH release was measured. (E) Cells were treated with increasing concentrations of Mdivi‑1, and cell viability was assessed by CCK‑8 assay. Data are presented as mean ± S.E.M., analyzed by one‑way ANOVA followed by Tukey’s post hoc test. ****P* < 0.001; ns, not significant.

## 2.4 Supplementary Figure 4

**Supplementary Figure 4.** **Mdivi‑1 rescues mitochondrial fission induced by MPP⁺ or *Atp13a2* knockdown in C8‑D1A cells.** (A) Representative immunoblots of p‑Drp1, Drp1, and Mfn2 in C8‑D1A cells treated with MPP⁺ in the presence or absence of Mdivi‑1 or LV‑*Atp13a2*‑siRNA (n = 4 per group). β‑actin served as a loading control. (B) Quantification of p‑Drp1 protein levels. (C) Quantification of Drp1 protein levels. (D) Quantification of Mfn2 protein levels. Data are presented as mean ± S.E.M., analyzed by two‑way ANOVA followed by Tukey’s post hoc test. **P* < 0.05, ****P* < 0.001; ns, not significant.

## 2.5 Supplementary Figure 5

**Supplementary Figure 5.** ***Atp13a2* overexpression attenuates MPP⁺‑induced mitochondrial fission and dysfunction in C8‑D1A cells.** (A) Representative immunoblots of ATP13A2, p‑Drp1, Drp1, OPA1, and Mfn2 in C8‑D1A cells treated with MPP⁺ (with or without Mdivi‑1 or LV‑*Atp13a2*‑OE; n = 4 per group). β‑actin served as a loading control. (B) Quantification of ATP13A2 protein levels. (C) Quantification of p‑Drp1 protein levels. (D) Quantification of Drp1 protein levels. (E) Quantification of OPA1 protein levels. (F) Quantification of Mfn2 protein levels. (G) Representative fluorescence images of TMRM staining (red) showing mitochondrial membrane potential. Nuclei were counterstained with DAPI (blue). Scale bar: 20 μm. (H) Quantification of TMRM fluorescence intensity (n = 4 per group). Data are presented as mean ± S.E.M.; statistical analysis was performed by two‑way ANOVA followed by Tukey’s post hoc test. **P* < 0.05, ***P* < 0.01, ****P* < 0.001; ns, not significant. Treatments are indicated below the blots and graphs.
